# Immunomodulatory and Antioxidant Effects of Polysaccharides from *Gynostemma pentaphyllum* Makino in Immunosuppressed Mice

**DOI:** 10.3390/molecules21081085

**Published:** 2016-08-19

**Authors:** Xiaoya Shang, Yu Chao, Yuan Zhang, Chengyuan Lu, Chunlan Xu, Weining Niu

**Affiliations:** 1 The Key Laboratory for Space Bioscience and Biotechnology, School of Life Sciences, Northwestern Polytechnical University, Xi’an 710072, Shaanxi, China; lcyecho@mail.ustc.edu.cn (C.L.); lx304319@163.com (C.X.); niuweining@126.com (W.N.); 2 Department of Orthopaedics, The First Affiliated Hospital of Xi’an Medical University, Xi’an 710077, Shaanxi, China; muyuchris@126.com; 3 Key Laboratory of High Altitude Environment and Related Illness of Tibet Autonomous Region, Department of Medicine, Xizang Minzu University, Xianyang 712082, Shaanxi, China; zhangyuan19880328@163.com

**Keywords:** *Gynostemma pentaphyllum* Makino, polysaccharide, in vivo, immunomodulatory, antioxidant

## Abstract

The immunomodulatory and antioxidant activities of crude polysaccharides extracted from *Gynostemma pentaphyllum* Makino (GPMPP) were investigated. GPMPP was composed of rhamnose, arabinose, xylose, mannose, glucose and galactose in the molar ratio of 1.39:3.76:1.00:1.64:4.98:5.88. In vivo studies showed GPMPP significantly increased the spleen and thymus indices, activated the macrophage phagocytosis and NK cells, and exhibited activity on none or Con A/LPS-stimulated splenocytes in a dose-dependent manner in C57BL/6 mice. Moreover, GPMPP elevated CD4^+^ T lymphocyte counts as well as the CD4^+^/CD8^+^ ratio dose-dependently, and it increased IL-2 level in the sera and spleen of Cy-immunosuppressed mice. Furthermore, GPMPP significantly increased the SOD, GSH-Px, T-AOC, GSH and CAT level, and decreased the MDA level. The results showed that GPMPP might play an important role in prevention of oxidative damage in immunological system. These findings indicate GPMPP has immunomodulatory activity in vivo and seems to be an effective natural immunomodulatory agent.

## 1. Introduction

It has been reported that xanthine oxidase, peroxisomes, inflammation processes, phagocytosis, as well as external factors such as smoking, environmental pollutants, radiation, drugs, and so on, can produce free radicals, which are a normal part of metabolism within the mitochondria [1]. Excessive amounts of reactive oxygen species (ROS) can become a source of tissue damage, because they are not counteracted by the antioxidant defenses of the cell. This can cause many diseases such as cancer, cardiovascular disease, neurological disorders, renal disorders, liver disorders, auto-immune deficiency diseases, inflammation, obesity, Alzheimer’s disease, and so on [2,3].

The immune system highly depends on accurate cell-cell communication for optimal function, and any damage to the signaling systems involved will cause an impaired immune responsiveness [4]. In order to defense against infection, phagocytes produce ROS and cause injury to target cells, which is a particular hazard to the immune system [5]. The immune cell functions are specially linked to ROS generation, and strongly influenced by the antioxidant/oxidant balance [6]. Therefore, adequate amounts of neutralizing antioxidants are required to prevent damage to the immune cells themselves. The antioxidants are central to the redox balance in the human body, and act synergistically but not in isolation [7,8]. They protect immune cells from oxidative stress and preserve their adequate function, maintaining immune cells in a reducing environment [9].

In the past several decades, polysaccharides isolated from botanical sources (mushroom, algae, lichens and higher plants) have attracted a great deal of attention in the biomedical area because of their broad spectrum of therapeutic properties and relatively low toxicity [10,11,12]. Polysaccharides from plants were considered to play an important role as dietary radical scavengers for the prevention of oxidative damage in living systems [13,14,15]. *Gynostemma pentaphyllum* Makino (*G. pentaphyllum* Makino), a perennial liana plant, grows widely in Southern China, Japan, India and Korea. It is a well-known edible and medicinal plant. *G. pentaphyllum* Makino has been reported to have antioxidant, immunopotentiating, anti-inflammatory, cholesterol-lowering, antitumor, cardiovascular, anti-hyperlipidemic and hypoglycemic effects [16,17,18]. Previous studies on polysaccharides extracted from *G. pentaphyllum* Makino focused on their antioxidant activities in vitro, which exhibited scavenging capacities against hydroxyl, peroxyl, DPPH and hydroxyl radicals [19,20]. Moreover, the polysaccharides from *G. pentaphyllum* Makino have antitumor and immunoregulatory activity in H_22_ tumor-bearing mice [21]. In our previous work, we found that the water-soluble polysaccharide extracted from *G. pentaphyllum* Makino showed the best ability in the inhibition of lipid spontaneous peroxidation, significant reducing power activity, DPPH and hydroxyl radicals scavenging ability and the inhibitory ability on lipid peroxidation induced by Fe^2+^-H_2_O_2_ [22]. However, very little is known about the antioxidant and immunomodulatory capacities of polysaccharides from *G. pentaphyllum* Makino in vivo. In the present study, the in vivo immunomodulatory and antioxidant activities of polysaccharides from *G. pentaphyllum* Makino were assessed.

## 2. Results and Discussions

### 2.1. Characterization of GPMPP

**Figure 1:**
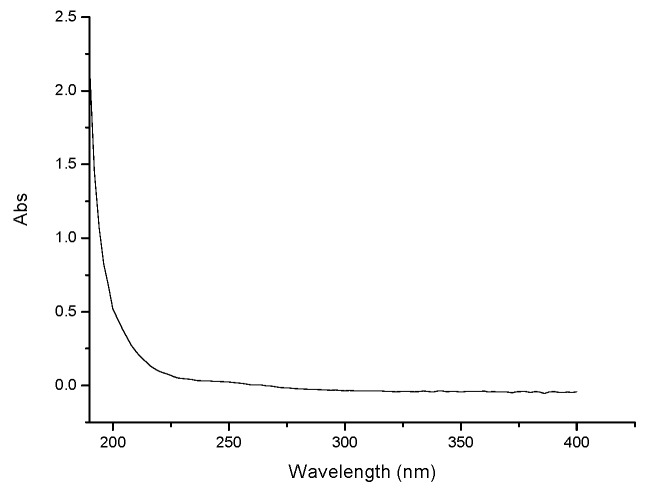
Ultraviolet absorption spectrum of GPMPP.

**Figure 2:**
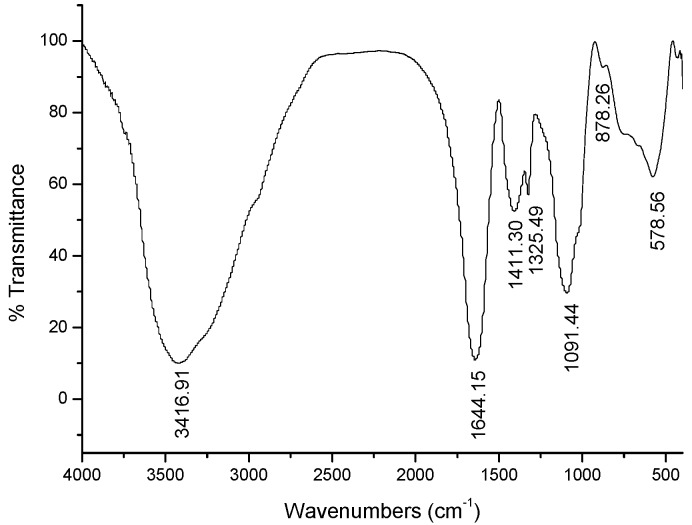
IR spectrum of GPMPP.

**Figure 3:**
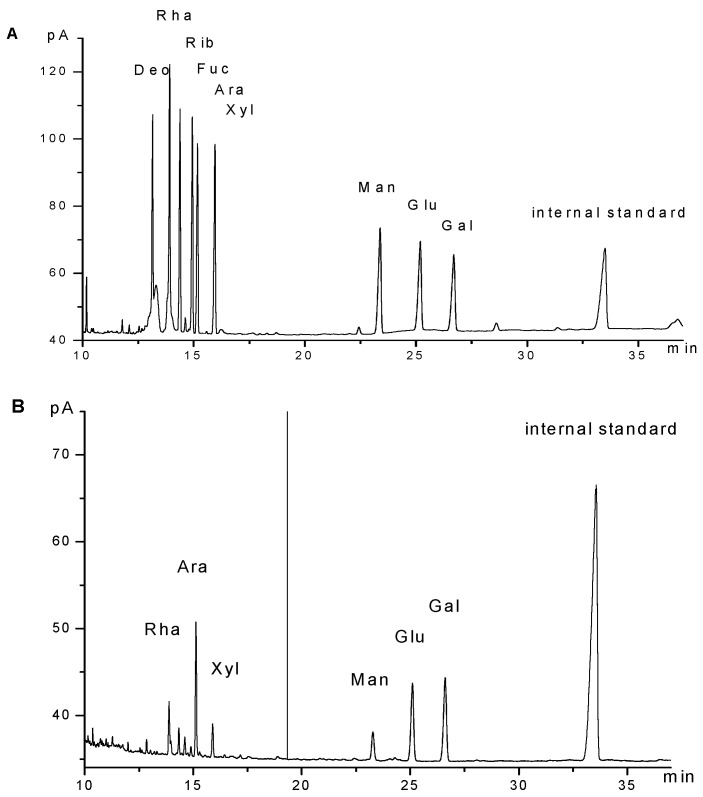
GC chromatograms of nine standard monosaccharides (**A**); component monosaccharides released from GPMPP; (**B**). d-deoxyribose (Deo), l-rhamnose (Rha), d-ribose (Rib), l-fucose (Fuc), d-arabinose (Ara), d-xylose (Xyl), d-mannose (Man), d-glucose (Glu), d-galactose (Gal).

A strong polysaccharide absorption was observed at 190 nm. Moreover, no absorption appeared at 260 or 280 nm in the UV spectrum, indicating the absence of nucleic acid and protein (Figure 1). GPMPP did not contain phenolic compounds, as detected by the ferric chloride color method. The IR spectrum of GPMPP displayed a characteristic intense broad stretching peak at around 3417 cm^−1^ due to hydroxyl groups (Figure 2). Further, an asymmetrical stretching peak can be found at 1644 cm^−1^ and a weak symmetrical stretching peak near 1430–1390 cm^−1^, suggesting the presence of carboxyl groups [23]. The absorption at 1091 cm^−1^ is related to C-O stretching vibrations. The absorption at 878 cm^−1^ may be indicative index of α–glycosidic linkages in GPMPP. From Figure 3, GPMPP was determined by GC of the corresponding acetylated monosaccharides to be composed of rhamnose, arabinose, xylose, mannose, glucose and galactose in the molar ratio of 1.39:3.76:1.00:1.64:4.98:5.88. Mannose, glucose and galactose are three predominant monosaccharides in GPMPP accounting for 78.43% of the total monosaccharides. The molecular weight of GPMPP was about 36.7 KDal by dynamic light scattering, and further structural analysis was performed by NMR, MS and so on.

### 2.2. Effects of GPMPP on Thymus and Spleen Indices in Cy-Immunosuppressed Mice

The spleen and thymus indices may reflect immune function and prognosis of an organism. Table 1 shows that the thymus indices of the animals treated with Cy at dose of 80 mg/kg/day for 3 days decreased significantly when compared with the normal control. The spleen and thymus indices of the animals treated with both GPMPP of 50, 150, or 250 mg/kg and Cy (80 mg/kg/day for 3 days) increased as compared with the animals treated with Cy alone. LH at dose of 10 mg/kg significantly raised the thymus indices compared with Cy. The spleen contains T and B cells, while the thymus is the organ containing T lymphocytes. The results indicated that the immune function was diminished when the animals were treated with Cy. Many polysaccharides have been found the similar results [24]. The findings suggested that GPMPP overcame the immunosuppressed action of Cy.

### 2.3. Effects of GPMPP on Macrophage Phagocytosis in Cy-Immunosuppressed Mice

The phagocytic index of the model group was significant lower than that of the normal group. The GPMPP effectively increased the phagocytic index of Cy-immunosuppressed mice dose-dependently. Higher concentration of GPMPP showed a greater phagocytic index, especially at the high dose of 250 mg/kg BW. This indicated that GPMPP played an important role in the initiation and regulation of nonspecific immune, and enhanced macrophage function in Cy-immunosuppressed mice.

### 2.4. Effects of GPMPP on Leukocytes and Bone Marrow Cells in Cy-Immunosuppressed Mice

The model group showed significantly reduced numbers of WBC and BMC. After treatment with low dose GPMPP, the numbers of WBC were restored to normal levels in Cy-immunosuppressed mice. Intermediate doses of GPMPP treatment significantly recovered of BMC counts compared with the model group. Levamisole hydrochloride (LH) treatment also increased WBC and BMC counts to the normal level. WBC are part of the immune system to fight infections, and BMC can produce and release more white blood cells in response to infections. Our results found that the results of WBC are associated with BMC. It has been confirmed that bone marrow produces white blood cells, which are necessary for a healthy immune system.

### 2.5. Effects of GPMPP on Lymphocyte Proliferation in Cy-Immunosuppressed Mice

**Figure 4:**
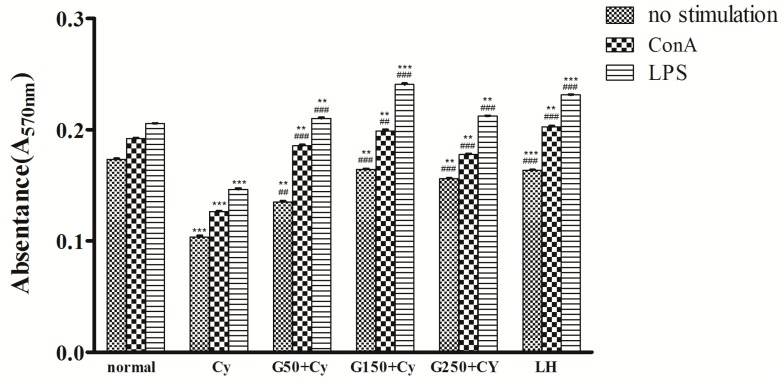
Effect of GPMPP on concanavalin A or lipopolysaccharide-induced lymphocyte proliferation in Cy-immunosuppressed mice. ** *p* < 0.01, *** *p* < 0.001, compared with normal control. ^##^ *p* < 0.01, ^###^ *p* < 0.001, compared with model control. Values are means ± SD, *n* = 10.

To further know the immunomodulatory activity of GPMPP, the effects of GPMPP on the proliferation of full splenic cells were investigated. One of the indicators of immunopotentiation is lymphocyte proliferation, which includes both T and B lymphocytes. It is known that ConA stimulates T cells, while LPS stimulates B cell proliferation [25]. In the current study, the splenocyte proliferation assays revealed that GPMPP had strong mitogenic potential to both T and B cells on the ConA-and LPS-activated splenocytes, respectively, indicating stimulatory effected on cell-mediated and humor-mediated immunity. ConA/LPS or non-stimulated splenocyte proliferation in the model group was significantly lower than that of the normal group. However, the treatment with GPMPP at the three tested doses resulted in a significant increase in ConA/LPS or no stimulation, and the intermediate dose of GPMPP treatment showed the best effects (Figure 4). T lymphocytes play a central role in cellular immunity, and B lymphocytes play a central role in humoral immunity. It had been reported that rice hull polysaccharides also stimulated the proliferation of T and B cells, but another report found that the polysaccharides from an herbal tea just enhance the proliferative ratio of ConA-induced lymphocytes [26,27]. It indicated that polysaccharides from Chinese herb and food have the potential ability to modulate lymphocyte proliferation, while the structure of polysaccharides played an important role in lymphocytes proliferation.

### 2.6. Effects of GPMPP on Serum Haemolysin in Cy-Immunosuppressed Mice

As shown in Table 2, the serum haemolysin levels in the model group was significant lower than in the normal group, but in all the testing groups they were higher than in the model group. Particularly, they almost reached a similar hemolysin level as the high dose GPMPP treatment and positive group, and both treatments exhibited the best effects. The effect of GPMPP on hemolytic activity was in a dose-dependent manner.

### 2.7. Effects of GPMPP on Natural Killer Cell Activity in Cy-immunosuppressed Mice

**Figure 5:**
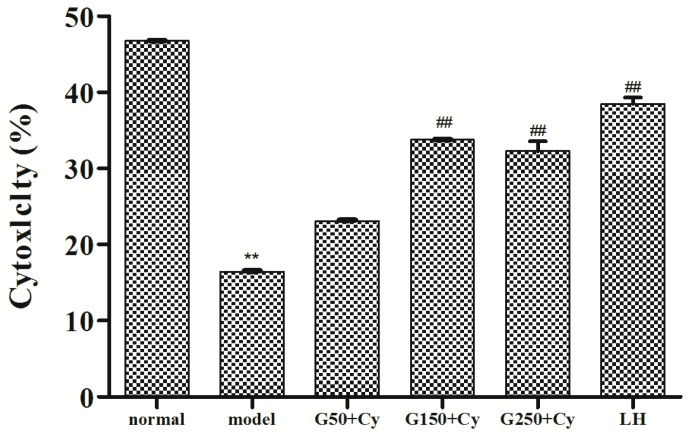
Effect of GPMPP on cytotoxicity of natural killer cells in Cy-treated mice. ** *p* < 0.01, compared with normal control. ^##^ *p* < 0.01, compared with model control. Values are means ± SD, *n* = 10.

The host defense against tumor cells and cells infected by some viruses strongly depends on NK cells, which are important in both innate and adaptive immune [28]. Figure 5 showed the effects of GPMPP on natural killer (NK) cells in Cy-immunosuppressed mice. It can be found that NK cell activities in the model group were significantly lower than that of the control group. However, NK cell activities of the 150 mg/kg BW and 250 mg/kg BW groups were significantly higher than that of the model group (*p* < 0.01). The results demonstrated that GPMPP could enhance the cytotoxicity of NK cells and improve the capacity of the host to fight against the virus-infected cells. Thus GPMPP has an important role in the non-specific immune system.

### 2.8. Effects of GPMPP on T-Lymphocyte Phenotyping in Cy-Immunosuppressed Mice

Two major functional subpopulations of T lymphocytes are CD4^+^ and CD8^+^ and the CD4^+^/CD8^+^ ratio reflects the biological activity of T lymphocytes. To evaluate the effects of GPMPP on the cellular immunity in Cy-immunosuppressed mice, the counts of CD4^+^ and CD8^+^ T lymphocytes from the spleens of Cy-immunosuppressed mice were measured using flow cytometry. The ratio of CD4^+^ to CD8^+^ reflects the biological activity of T lymphocyte. As shown in Table 3, the CD4^+^/CD8^+^ ratio of model group in splenocyte suspensions remarkably decreased compared to the normal group. With the stimulation of GPMPP at different dosages, the CD4^+^/CD8^+^ ratio was increased to the normal level. Furthermore, at a dose of 150 mg/kg BW in GPMPP groups, the ratios of CD4^+^/CD8^+^ approached that of the positive control group, suggesting that the ability of GPMPP in balancing the T lymphocyte subsets was similar to that of LH. The results from the present investigation showed that the CD4^+^/CD8^+^ ratio was increased to the normal level by GPMPP. The enhanced CD4^+^/CD8^+^ ratio could help the host improve the immune response against foreign antigens and pathogens. CD4^+^ T cells contain Th-1 and Th-2and generally function as T helper (Th) cells, while CD8^+^ T cells generally function as T cytotoxic (Tc) cells [29].

### 2.9. Effects of GPMPP on IL-2 Expression in Cy-Immunosuppressed Mice

As shown in Table 4, IL-2 levels in sera and spleen in the model group were lower than those of the control group. The levels of IL-2 in sera and spleen were significantly increased after treatment with different doses (Table 4), indicating GPMPP can regulate the immune activity by stimulating the IL-2 in sera and spleen. The IL-2 content was close to the positive control group at a dose of 250 mg/kg BW GPMPP. Compared with the normal and model groups, GPMPP treatment dose-dependently enhanced the levels of IL-2 in the GPMPP treatment groups. CD4^+^ T cells produce different cytokines and the secretion of IL-2 belongs to a Th-1 type cellular response [30]. IL-2 can activate T cell proliferation and NK cell activities. In this paper, we found that GPMPP treatment significantly increased the levels of IL-2 in sera (*p* < 0.001), which indicated that the immunomodulatory activity of mice was obviously improved after administration of GPMPP. Previous studies have found that several polysaccharides from plants could increase CD4^+^/CD8^+^ ratio and induce production of cytokines including IL-2 [31,32]. The present study results are in agreement with previous results, which demonstrated that GPMPP administration might improve cellular immune function of immunosuppressed mice.

### 2.10. Effects of GPMPP on Activities of Antioxidant Enzymes in Cy-Immunosuppressed Mice

**Figure 6:**
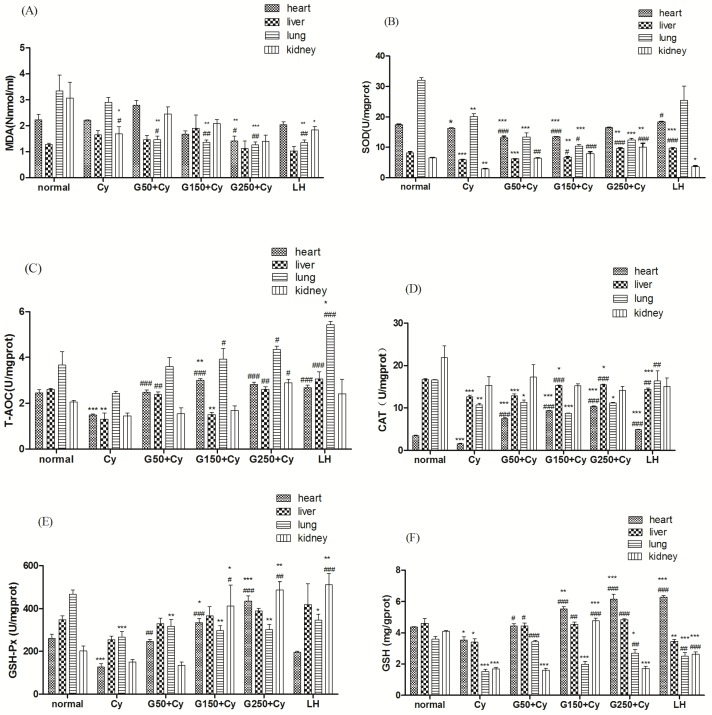
Effect of GPMPP on the (**A**) MDA level, and the activities of (**B**) SOD; (**C**) T-AOC; (**D**) CAT; (**E**) GSH-Px and (**F**) GSH in the heart, liver, lung and kidney of Cy-immunosuppressed mice. * *p* < 0.05, ** *p* < 0.01, *** *p* < 0.001, compared with normal control. ^#^ *p* < 0.05，^##^ *p* < 0.01，^###^ *p* < 0.001, compared with model control. Values are means ± SD, *n* = 10.

Although GPMPP displayed in vivo immunomodulatory activity, the exact mechanism of its action isn’t well understood. The endogenous genotoxic product of enzymatic and oxygen radical-induced lipid peroxidation is malondialdehyde (MDA), which exists in DNA isolated from healthy human beings [33]. When the animals were treated with Cy, the MDA level in the heart and liver changed a little (Figure 6A). In the Cy-induced group treated with a GPMPP dose of 50 mg/kg, MDA in the lungs decreased significantly. At the middle and high doses of GPMPP, MDA in the heart, lung and kidney decreased, especially in lung. When the GPMPP dose is 250 mg/kg, MDA in the heart, liver, lung and kidney reached the level of the LH group. Superoxide dismutase (SOD) protects against oxidative processes initiated by superoxide anion [34]. The activity of SOD in the heart, liver, lung and kidney of Cy-treated animals decreased significantly when compared with the normal control, especially in the liver (Figure 6B). In the animals treated with different doses of GPMPP, the activity of SOD in the heart, liver and kidney significantly increased as compared with the Cy control. The activity of SOD in the lung significantly raised only with 150 mg/kg dose of GPMPP compared with the Cy control. Figure 6C,D show the activities of T-AOC and CAT in the heart, liver, lung and kidney of each group. T-AOC and CAT decreased remarkably (*p* < 0.001 and *p* < 0.01, respectively) with Cy-treatments in the heart and liver, except in the kidney. The GPMPP administration greatly elevated the T-AOC in the heart, liver, lung and kidney. The level of T-AOC at the GPMPP dose of 250 mg/kg significantly increased in all tested organs, reached even higher levels than the normal group. Compared with the control group, different doses of GPMPP significantly raised the CAT activity in the heart and liver compared with the model group (*p* < 0.001). The heart and liver CAT activity in GPMPP-treated mice at the dose of 250 mg/kg reached 10.37 ± 0.08 U/mg protein and 15.47 ± 0.06 U/mg protein, respectively. The findings indicated that GPMPP at high dose can compare with LH, hich showed that it has the ability to improve the immunomodulatory activity in normal mice and treat Cy-immunosuppressed mice. The main biological role of glutathione peroxidase (GSH-Px) is to protect the organism from oxidative damage [35]. Glutathione (GSH) prevents damage to important cellular components caused by reactive oxygen species such as free radicals and peroxides [36]. As shown in Figure 6E,F, the Cy group had lower GSH-Px and GSH activities compared with the normal group, and the difference in lung is significant (*p* < 0.001). The GPMPP administration increased the GSH-Px in liver and kidney of the mice treated with Cy, moreover significantly enhanced in heart and lung (*p* < 0.001 and *p* < 0.01, respectively), and significantly increased the GSH in heart, liver, lung and kidney. When the dose of GPMPP reached 250 mg/kg, the activities of GSH-Px and GSH in heart reached the best level, even better than that of LH. GPMPP showed better effects on the GSH-Px activity in heart and kidney than in liver and lung. Figure 6E,F indicated that GPMPP can improve the activity of GSH-Px and GSH in mice, which showed stronger effects on increase of the GSH-Px and GSH levels than LH treatment.

Glutathione peroxidase (GSH-Px), superoxide dismutase (SOD) and catalase (CAT) are important enzymes, which detoxify lipid hydroperoxides, superoxide radicals and hydrogen peroxide, respectively [37]. If our bodies are under oxidative stress, one or more antioxidant enzymes decrease [38]. In Cy-treated control mice, we found that the activities of GSH-Px, SOD and CAT were significantly lower than in the normal control mice. This suggested that there were higher levels of lipid hydroperoxides, superoxide radicals and hydrogen peroxide in mice treated with Cy, leading to increased ROS levels. This indicated that Cy could reduce the antioxidant enzyme activities. However, after being treated with GPMPP, the activity of GSH-Px, SOD and CAT increased in a dose-dependent manner. The results suggest that the lipid peroxidation inhibition of GPMPP might be due to its effects on the antioxidant enzyme system. These findings indicated that GPMPP could act against the immune inhibition induced by Cy, suggesting that GPMPP can protect the immune organs by increasing the activities of antioxidant enzymes.

## 3. Experimental Section

### 3.1. Materials

*G. pentaphyllum* Makino was obtained from Pingli County of Shaanxi Province, China. d-Deoxyribose (Deo), l-rhamnose (Rha), d-ribose (Rib), d-arabinose (Ara), l-fucose (Fuc), d-xylose (Xyl), d-mannose (Man), d-glucose (Glu), d-galactose (Gal) and bovine serum albumin (BSA) were purchased from Sigma (St. Louis, MO, USA). RPMI-1640 medium, fetal bovine serum (FBS), 0.25% trypsin ethylene diaminetetraacetic acid (EDTA), concanavalin A (Con A) and lipopolysaccharide (LPS) were purchased from Gibco/Life Technologies (Grand Island, NY, USA). 3-(4,5-Dimethylthiazol-2-yl)-2,5-diphenyltetrazolium bromide (MTT) was obtained from Sigma-Aldrich. Dimethyl sulfoxide (DMSO) and 2,4-dinitrofluorobenzene (DNFB) were purchased from Xi’an Xintai Reagent Company (Xi’an, China). Cyclophosphamide (Cy) and levamisole hydrochloride (LH) were provided by The First Affiliated Hospital of Xi’an Medical University. Assay kits for malondialdehyde (MDA), superoxide dismutase (SOD), catalase (CAT), glutathione peroxidase (GSH-Px), total antioxidant capacity (T-AOC), reduced glutathione (GSH), and protein content were obtained from the Nanjing Jiancheng Bioengineering Institute (Nanjing, China). C57BL/6 mice (8 weeks old, 20 ± 2 g) were provided by the Animal Experimental Center of Xi’an Jiaotong University, Xi’an, China.

### 3.2. Preparation of the Crude Polysaccharides from G. pentaphyllum Makino

The dried whole *G. pentaphyllum* Makino (200 g) was pretreated with 95% ethanol (1 L) at 50 °C three times to remove lipids and then the organic solvent was volatilized at room temperature and pretreated dried *G. pentaphyllum* Makino was obtained. The pretreated dried *G. pentaphyllum Makino* was extracted with distilled water and the supernatant was collected. The supernatant was concentrated, and precipitated with ethanol (1:4, *v*/*v*). The mixture was kept at 4 °C for 12 h to precipitate the polysaccharides. The precipitate that formed was collected by centrifugation at 5000 rpm and repeatedly washed sequentially with minimal amounts of ethanol, acetone and ether, respectively. Then the precipitate was dissolved in distilled water, treated with 30% hydrogen peroxide at 50 °C for 8 h and dialyzed against still distilled water for 48 h, changing the distilled water every 4 h with dialysis tubing (molecular weight cut-off, 3500 Da) to remove low-molecular weight matter (e.g., chromones and anthranoids), and then concentrated and precipitated with 4-fold volumes of 95% ethanol to obtain the polysaccharides. The precipitate formed was collected by centrifugation at 5000 rpm and repeatedly washed sequentially with the least possible amount of ethanol, acetone and ether, respectively. Then the precipitate was dried at reduced pressure, and the dried white powder obtained was named GPMPP (10.59 g). Total sugar content of the polysaccharide was determined by the phenol-sulfuric acid method, using glucose as the standard. 

### 3.3. Characterization of GPMPP

GPMPP was dissolved in distilled water and forced through a 0.45 μm filter membrane to obtain a 1 mg/mL polysaccharide solution. The solution was then scanned in the wavelength range of 190–400 nm using a UV spectrophotometer (U-3310, Hitachi, Tokyo, Japan). The IR spectrum of GPMPP was determined using a Fourier Transform infrared spectrophotometer (FT-IR-8400S, Shimadzu, Kyoto, Japan) over the range of 4000–400 cm^−1^ with a resolution of 4 cm^−1^. For monosaccharide composition analysis, GPMPP (10 mg) was dissolved in 10 mL 2 mol/L trifluoroacetic acid and hydrolyzed at 100 °C for 4 h in a sealed glass tube. The hydrolysates were converted to acetylated aldononitrile derivatives according to conventional protocols and analysed by gas chromatography (GC) on an Agilent 6890 system GC (Agilent Technologies, Palo Alto, CA, USA) with myo-inositol as the internal standard The Agilent 6890 system used above was fitted with a DB-1701 capillary column (30 m × 0.25 mm × 0.25 μm) and a flame-ionisation detector (FID). Alditol acetates of the nine standard monosaccharides (d-deoxyribose, l-rhamnose, d-ribose, d-arabinose, l-fucose, d-xylose, d-mannose, d-glucose, d-galactose) were prepared and subjected to GC analysis separately. The operation was performed under the following conditions: H_2_, 40 mL/min; air, 400 mL/min; N_2_, 1 mL/min; injection temperature, 280 °C; detector temperature, 280 °C. The oven temperature programmer was 3 min at 110 °C, 20 °C/min to 210 °C, and finally holding for 30 min at 210 °C. The temperature of the injector and detector was 280 °C. Injections were made in the splitless mode.

### 3.4. Animals and Experimental Design

Male C57BL/6 mice (8 weeks old, 20 ± 2 g) were housed in open top cages, maintained under the control conditions of temperature (22 ± 1 °C) and humidity (50% ± 5%), with a 12 h light-dark cycle for acclimatization. They were provided with water and mouse chow ad libitum. All experimental animals were overseen and approved by the Animal Care and Use Committee of our Institute before and during experiments. Mice were randomly divided into six groups (each group *n* = 10). One group of healthy mice was used as normal controls without Cy-treatment. From day 1 to 3, the other five groups of mice were subjected to immunosuppression by administration of Cy (80 mg/kg/day) intraperitoneally. One group of those Cy-treated mice was used as a model group. From day 4 to day 18, the mice were administered the following treatments: Group I (normal): normal control (physiological saline solution); Group II (Cy): model control (physiological saline solution); Group III (G50 + Cy): Cy + low-dose GPMPP (50 mg/kg body weight GPMPP); Group IV (G150 + Cy): Cy + intermediate-dose GPMPP (150 mg/kg body weight GPMPP); Group V (G250 + Cy): Cy + high-dose GPMPP (250 mg/kg body weight GPMPP); Group VI (LH): positive control (10 mg/kg body weight levamisole hydrochloride). Cy (0.2 mL) was administered via intraperitoneal injection. The other treatments were administered via gavage in 0.2 mL solutions. Twenty-four hours after the last administration of GPMPP, all animals were weighed and sacrificed by cervical dislocation. The heart, liver, lung, kidney, spleen, and thymus were excised from the animals and weighted immediately. The spleen and thymus indices were calculated according to the following formula: thymus or spleen index (mg/g) = (weight of thymus or spleen/body weight). The collected tissues and serum were stored in –80 °C for further analysis.

### 3.5. Macrophage Phagocytosis Assay

The phagocytosis function of monocytes was assessed through a carbon clearance test according to the method of Hua et al. with minor modifications [39]. After 18 days of oral administration of GPMPP, Cy or saline, India ink (100 μL/10 g body weight) was injected via the tail vein. Blood (20 μL) was collected by retro-orbital puncture at 2 min (T_1_) and 10 min (T_2_) after injection and then added to 2 mL of 0.1% Na_2_CO_3_. The absorbance at 600 nm (A_600_) of the 2 min (A_1_) and 10 min (A_2_) blood samples was measured on a UV-visible spectrophotometer (U-3310, Hitachi) with 0.1% Na_2_CO_3_ as the blank. The body, liver and spleen weights were measured after the mice were sacrificed by cervical dislocation. The phagocytic index (α) was calculated as follows: Rate of carbon clearance (κ) = (lg A_1_ − lg A_2_)/(T_1_ − T_2_), Phagocytic index (α) = [body weight/(liver weight + spleen weight)] × K^1/3^, where A_1_ is the absorbance at 2 min; A_2_ is the absorbance at 10 min; T_1_ is the time of blood collection at 2 min; T_2_ is the time of blood collection at 10 min.

### 3.6. Measurements of Leukocytes and Bone Marrow Cells

Peripheral blood samples of each group were collected in heparinized tubes. Leukocytes were counted with an automated chemical analyzer (7600; Hitachi). Bone marrow cell (BMC) suspensions were prepared by flushing a femur with serum-free RPMI-1640 media through syringe needles several times. Total numbers of BMC were counted under light microscopy.

### 3.7. Splenocyte Proliferation Assay

The mice spleens were removed aseptically at the end of experiment from the sacrificed mice in 0.1 M cold PBS and passed through a sieve of 200 mesh size to make single-cell suspensions. After treatment with erythrocyte lysis buffer, the spleen cells were resuspended to a final density of 1 × 10^6^ cells/mL in RPMI 1640 medium supplemented with 10% newborn bovine serum, 100 U/mL penicillin and 100 μg/mL streptomycin. Spleen cell suspension (200 μL) was added to a 96 well microtiter plate with either RPMI 1640 medium, LPS (final concentration 10 μg/mL) or Con A (final concentration 5 μg/mL), then incubated for 48 h at 37 °C with 5% CO_2_. Each well was further incubated for 4 h with 20 μL MTT (3-[4,5-dimethylthiazol-2-yl]-2,5 diphenyltetrazolium bromide; 5 μg/mL). The plates were centrifuged at 200 g for 15 min and the MTT medium was removed. A volume of 150 μg/mL DMSO solution was added to each well, and shaken until all crystals dissolved. The absorbance at 570 nm was measured using a microplate reader (BioTekSynergy HT, Winooski, VT, USA).

### 3.8. Serum Haemolysin Assay

Serum haemolysin was evaluated using a previously described method with modification [40]. On the fifth and thirteenth day of GPMPP or vehicle administration, the control and experimental mice were immunized twice by intraperitoneal injection of 0.2 mL sheep erythrocyte suspension (SRBC, 2%, *v*/*v*). Five days later, serum was collected through the eye orbit and then diluted with PBS according to multiple proportions. At 1 h after the last inspection, the blood samples were collected through the eye orbit of the mouse. The blood samples were left to stand for 1 h, and then centrifuged at 2000 rpm for 15 min to separate the serum. The serum was diluted 100 times with saline. Then 100 μL of diluted serum was transferred to a hemagglutination microplate, and 100 μL of SRBC (0.5%, *v*/*v*) was added, followed by 100 μL guinea pig serum in sequence. Distilled water or PBS buffer was included as positive and negative controls for haemolysis. The diluted serum was substituted with PBS as a blank control. The mixtures were incubated at 37 °C for 1h and then centrifuged at 2000 rpm for 10 min. 150 μL of each supernatant was transferred to a flat- bottom microplates and the optical density (OD) at 415 nm was measured using a microplate reader (BioTek Synergy HT). Haemolytic activities by saponins were calculated based on the following: percentage of haemolysis = [(A_s_ − A_n_)/(A_p_ − A_n_)]× 100%. A_s_, A_n_ and A_p_ represented absorbance value of samples, negative control and positive control, respectively. Each sample was tested in triplicate.

### 3.9. Natural Killer Cell Activity Assay

Natural killer (NK) cell activity was evaluated using the methio described by. Chalamaiah d [41]. YAC-1 cells were used as target cells, and the concentration was 4.0 × 10^4^ cell/well (100 μL/well) in 96-well culture plates. Splenocytes were used as effector cells, and 100 μL splenocytes (2 × 10^6^ cells/mL) were added to each test well to reach an effector-target (E/T) ratio of 50:1. The plates were incubated for 20 h at 37 °C in a 5% CO_2_ atmosphere. Then 10 μL MTT (5 mg/mL) was added to each well and the plate was incubated for another 4 h under the same conditions. MTT assay was performed as mentioned previously. Three types of control measurements were performed: target cells control (YAC-1 cells without splenocytes), blank control (only medium without YAC-1 cells and splenocytes), and effector cells control (splenocytes without YAC-1 cells). NK cell activity was calculated using the following equation:

(1) $$NK\;cell\;activity\;\left(\%\right)=\;\frac{OD_{T}-\left(OD_{S}-OD_{E}\right)}{OD_{T}}\times 100\%$$

where OD_T_ = optical density value of the target cells control, OD_S_ = optical density value of the test samples, OD_E_ = optical density value of the effector cells control.

### 3.10. T-Lymphocyte Phenotyping Analysis

Single splenocytes cell suspension was prepared as described above. Splenocytes (1 × 10^6^ cells/mL) were incubated with 10 μL of either FITC-conjugated anti-CD3 or PE-conjugated anti-CD4 antibodies for 30–60 min at 4 °C. Cells were then washed twice with PBS and resuspended in 1% paraformaldehyde (PFA). The counts of CD4^+^ and CD8^+^ T lymphocytes were determined by flow cytometer (BD Biosciences, Bedford, MA, USA). The results were expressed in percentage of CD4^+^/CD8^+^ expression.

### 3.11. IL-2 Expression Assay

IL-2 contents in serums collected from Cy-treated mice and supernatants of splenocyte were determined with a mouse IL-2 ELISA kit (eBioscience, San Diego, CA, USA). All procedures were performed according to manufacturer′s instructions.

### 3.12. Biochemical Assay

A small portion of murine heart, liver, lung or kidney was removed and kept on ice and homogenized with 0.1 g/mL wet weight of ice-cold isotonic physiological saline. The samples were centrifuged at 3000 rpm/min at 4 °C for 15 min, and the supernatants were used to measure the protein, MDA, SOD, CAT, T-AOC, GSH-Px and GSH levels. The activity of SOD was measured with the xanthine oxidase method. The content of MDA was detected with barbituric acid reaction chromometry. The GSH-Px activity was measured with a modified glutathione exhaustion assay. The content of GSH was measured with a colorimetric method. The level of T-AOC was measured by the ferric reducing/antioxidant power assay method. The CAT activity was measured with the ammonium molybdate method.

### 3.13. Statistical Analysis

The results were expressed as the mean ± standard deviation (SD). The results were analyzed by one-way analysis of variance (ANOVA) test using the Statistical Package of the Social Science (SPSS 17.0) program (IBM, New York, NY, USA). A value of *p* < 0.05 was regarded as statistical significance.

## 4. Conclusions

Our study demonstrates that immune-suppression is associated with decreased levels of enzymatic antioxidants. GPMPP markedly enhanced the thymus and spleen indices in Cy-immunosuppressed mice, cellular immune response and enzymatic antioxidant activities, which strongly support the hypothesis that GPMPP can play an important role in preserving the immune function by reducing oxidative stress in mice.

## Figures and Tables

**Table 1:** Effects of GPMPP on spleen, thymus and phagocytic index of Cy-immunosuppressed mice.

Group	Dose (mg/kg BW)	Spleen Index (mg/g)	Thymus Index (mg/g)	Phagocytic Index (α)
Normal control	--	6.23 ± 0.87	1.55 ± 0.07	5.212 ± 0.03
Model control (Cy)	80	4.41 ± 0.92 **	1.26 ± 0.09 **	3.983 ± 0.02 ***
GPMPP	50 + Cy	5.27 ± 1.69	1.28 ± 0.09	4.771 ± 0.04 ^###^
	150 + Cy	6.14 ± 1.24 ^##^	1.52 ± 0.18 ^##^	5.096 ± 0.02 **^###^
	250 + Cy	6.45 ± 1.38 ^###^	1.49 ± 0.20 ^##^	5.458 ± 0.04 **^###^
Positive control (LH)	10 + Cy	6.63 ± 1.26	1.57 ± 0.11	5.282 ± 0.04 ^###^

** *p* < 0.01, *** *p* < 0.001, compared with normal control. ^##^ *p* < 0.01, ^###^ *p* < 0.001, compared with model control. Values are means ± SD, *n* = 10.

**Table 2:** Effects of GPMPP on numbers of white blood cells (WBC) and bone marrow cells (BMC), and haemolysin in sera in Cy-immunosuppressed mice.

Group	Dose (mg/kg BW)	WBC (10^6^/mL)	BMC (10^6^/mL)	Serum Haemolysin (HC_50_)
Normal control	--	5.7 ± 0.8	4.1 ± 0.8	0.393 ± 0.030
Model control (Cy)	80	3.3 ± 0.7 *	2.1 ± 0.3 **	0.259 ± 0.023 **
GPMPP	50 + Cy	4.0 ± 0.8 ^##^	2.5 ± 0.4	0.291 ± 0.018 ^#^
	150 + Cy	4.5 ± 0.9 ^##^	3.6 ± 0.6 ^###^	0.399 ± 0.013 ^##^
	250 + Cy	4.9 ± 1.0 ^##^	3.9 ± 0.3 ^##^	0.422 ± 0.010 ^##^
Positive control (LH)	10 + Cy	5.6 ± 0.5 ^##^	4.0 ± 0.9 ^##^	0.426 ± 0.013 ^##^

* *p* < 0.05, ** *p* < 0.01, compared with normal control. ^#^ *p* < 0.05, ^##^ *p* < 0.01, ^###^ *p* < 0.001, compared with model control. Values are means ± SD, *n* = 10.

**Table 3:** Effect of GPMPP on T lymphocyte subsets from spleen in Cy-immunosuppressed mice.

Group	Dose (mg/kg BW)	CD4^+^ (%)	CD8^+^ (%)	CD4^+^/CD8^+^
Normal control	--	50.10 ± 3.53	34.57 ± 2.69	1.45 ± 0.12
Model control (Cy)	80	44.44 ± 3.47 *	38.68 ± 3.40	1.15 ± 0.03 **
GPMPP	50 + Cy	45.69 ± 2.67	30.19 ± 3.56 ^###^	1.38 ± 0.1 ^#^
	150 + Cy	55.07 ± 4.8 *^#^	34.97 ± 3.41 ^##^	1.58 ± 0.07 *^##^
	250 + Cy	51.80 ± 1.49 *^#^	38.45 ± 4.45 ^#^	1.45 ± 0.1 *^#^
Positive control (LH)	10 + Cy	42.58 ± 4.23	27.36 ± 5.59	1.58 ± 0.17^##^

* *p* < 0.05, ** *p* < 0.01, compared with normal control. ^#^ *p* < 0.05, ^##^ *p* < 0.01, ^###^ *p* < 0.001, compared with model control. Values are means ± SD, *n* = 10.

**Table 4:** Effects of GPMPP on IL-2 impression in sera and spleen in Cy-immunosuppressed mice.

Group	Dose (mg/kg BW)	IL-2 (pg/mL)	
		in Sera	in Spleen
Normal control	--	0.361 ± 0.014	0.278 ± 0.012
Model control (Cy)	80	0.334 ± 0.034	0.261 ± 0.015
GPMPP	50 + Cy	0.427 ± 0.034 *^##^	0.383 ± 0.021 ***^###^
	150 + Cy	0.450 ± 0.018 ***^###^	0.376 ± 0.026 ***^###^
	250 + Cy	0.561 ± 0.025 ***^###^	0.487 ± 0.031 ***^###^
Positive control (LH)	10 + Cy	0.523 ± 0.027 ***^###^	0.463 ± 0.017 ***^###^

* *p* < 0.05, *** *p* < 0.001, compared with normal control. ^##^ *p* < 0.01, ^###^ *p* < 0.001, compared with model control. Values are means ± SD, *n* = 10.
